# Sociodemographic differences in the understanding of front-of-pack nutrition labeling, perception of healthfulness, and food purchase intention in Brazil

**DOI:** 10.29219/fnr.v70.13219

**Published:** 2026-07-03

**Authors:** Hellen Batista de Jesus Paviotti, Sarah Morais Senna Prates, Ilka Afonso Reis, Lucilene Rezende Anastácio

**Affiliations:** Department of Food Science, School of Pharmacy, Federal University of Minas Gerais (UFMG), Belo Horizonte, MG, Brazil

**Keywords:** consumer behavior, nutrition labeling, sociodemographic factors, purchase intention, perception

## Abstract

While nutrition claims draw attention to desirable nutrient qualities, the Brazilian Front-of-Pack Nutrition Labeling (FOPNL) system serves a contradictory purpose by signaling excessive levels of added sugar, saturated fat, and sodium.

Besides labeling, sociodemographic factors may influence the understanding of nutritional information and food choices. This experimental, controlled, and randomized study used secondary data derived from previously published research to evaluate the impact of sociodemographic variables, including region, sex, age, education, and income. Specifically, we analyzed how these factors influence the understanding of nutritional information, the perception of healthfulness, and the purchase intention of products with different FOPNL models and nutrition claims.

A sample of 720 Brazilian adults completed an online questionnaire, being randomly assigned to one of four FOPNL conditions: control (without FOPNL), octagon, triangle, or magnifying glass. Participants evaluated 12 label panels in a 3×2×2 factorial design, considering 1) food category, 2) number of nutrients in excess and 3) presence/absence of nutrition claims.

Understanding of nutritional information was measured as the ability to correctly identify nutrients in excess using a generalized linear model with binary logistic regression. Perception of healthfulness and purchase intention were assessed on a 7-point scale using mixed analysis of variance models, with the sociodemographic variables (region, sex, age, education, and income) as fixed effects and participants as random effects.

The results indicate that sociodemographic variables did not significantly affect the understanding of nutritional information. However, participants aged 25 to 34 and male participants reported higher perception of healthfulness and greater purchase intention compared to other groups. These findings suggest that, although the provision of nutritional information on packages supports informed food choices across diverse sociodemographic contexts, age and sex specifically influenced how consumers perceived product healthfulness and their likelihood of purchasing the presented items in this sample.

## Popular scientific summary

- Front-of-Pack Nutrition Labeling (FOPNL) highlights the presence of excess sugar, saturated fat, and sodium in foods, contrasting with nutrition claims, which often emphasize positive aspects of products. This experimental study with 720 Brazilians evaluated whether sociodemographic factors influence the understanding of nutritional information, perception of healthfulness, and purchase intention. No effect was found on the understanding of nutritional information; however, men and adults aged 25–34 showed a higher perception of healthfulness and greater purchase intention.

The current epidemiological scenario in Brazil is marked by a nutritional transition characterized by increased consumption of ultra-processed foods, as defined by the NOVA classification. These products are industrial formulations predominantly composed of substances extracted or derived from foods, with little or no whole foods, and typically contain additives to enhance sensory properties (1–3). In general, they have an unfavorable nutritional profile, characterized by high levels of added sugars, sodium, and saturated fat. Excessive intake of these nutrients is among the main dietary risk factors for the development of non-communicable diseases (NCDs) (4).

Therefore, with the aim of promoting healthier eating habits, nutrition labeling has emerged as a public health instrument, as it provides information about the nutritional content of food products purchased and consumed by the population (5, 6). However, difficulty in understanding and interpreting nutrition labeling can negatively affect consumer purchasing decisions and food choices (7).

Front-of-pack nutrition labeling (FOPNL) aims to facilitate nutritional understanding by bridging the gap resulting from the limitations consumers face when interpreting the quantitative nutrient declarations typically found on the back of food packaging (8). FOPNL consists of labels displayed on the main panel of product packaging that provide quick and simple information about the nutritional quality of foods (9).

Multiple FOPNL systems coexist worldwide, differing substantially in format and purpose, and encompassing interpretive, semi-interpretive, non-interpretive, and hybrid models (3, 9). Interpretive models integrate multiple criteria to provide an overall assessment of the healthfulness of a product, such as ranking systems and health endorsement logos. Semi-interpretive models present information on specific nutrients using symbols, qualitative descriptors, or color coding to facilitate comprehension, as seen in traffic light systems and warning labels. In contrast, non-interpretive models display numerical nutrient values without offering evaluative judgments or interpretive aids, such as the Guideline Daily Amounts (GDA). Hybrid models combine non-interpretive features with interpretive or semi-interpretive elements, including color-coded GDA schemes and the Health Star Rating system (3, 5). Furthermore, while some jurisdictions allow voluntary adoption of FOPNL systems, others mandate their implementation.

In Brazil, the mandatory FOPNL system employs a black magnifying glass symbol accompanied by the expression ‘high in’ to indicate products containing high amounts of added sugars, saturated fat, or sodium (8, 10). Unlike countries such as Mexico (11) and Argentina (12), Brazil does not prohibit the use of nutrition claims on products bearing warning labels, provided that such claims do not refer to the nutrients highlighted by the FOPNL system (8, 10). Countries that prohibit the joint presentation of this information do so to prevent consumer confusion, as individuals may have difficulty integrating both the positive attributes of a product and its nutritional disadvantages into an informed purchase decision (13). In this context, nutrition claims emphasize positive attributes of food composition and may enhance product appeal, perception of healthfulness, and purchase intention; however, they may also hinder accurate interpretation of nutritional information (7, 14, 15, 16, 17).

In addition to the elements that make up nutrition labeling, such as FOPNL and nutrition claims, other variables, including sociodemographic factors such as region, income, sex, age, and education, may also influence consumer perceptions and choices (18, 19). Higher levels of education and income have been associated with greater use and understanding of nutrition labeling (19, 20), but not necessarily with healthier food choices (21). On the other hand, increases in the prices of natural and minimally processed foods may reflect lower purchasing power among low-income populations and a greater likelihood of consuming ultra-processed and refined products (22–24).

In terms of sex, women are more likely to be concerned about health and healthy eating. They generally exhibit healthier eating patterns, such as higher consumption of natural foods and greater regularity in meal consumption, whereas men tend to prioritize specific flavors and exhibit distinct meal-related behaviors (25).

Investigating this topic is essential to provide evidence on how sociodemographic characteristics affect the perception of FOPNL systems, particularly in Brazil, which has recently implemented the magnifying glass model. This research contributes to the scientific literature by examining how sociodemographic characteristics of Brazilian adults relate to their interpretation of nutritional information. Accordingly, we tested the hypothesis that sociodemographic variables (age, sex, education, region, and income) significantly influence the understanding of nutritional information, perception of healthfulness, and purchase intention among consumers for products featuring different FOPNL models and nutrition claims.

## Methods

**Fig. 1:**
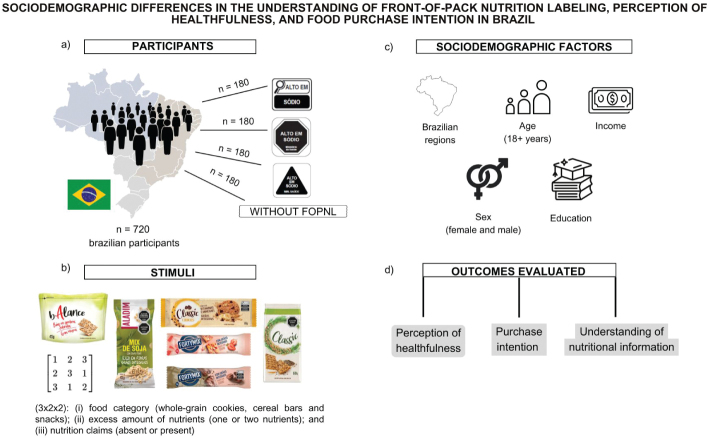
Overview of the study design.

This is an experimental, controlled and randomized study, using secondary data from a previously published study (14). The primary study was conducted through an online questionnaire and evaluated the effect of different FOPNL models (octagon, triangle and magnifying glass) (Fig. 1a), in the presence or absence of nutrition claims, on the understanding of nutritional information, perception of healthfulness, and purchase intention among Brazilian consumers in relation to supposedly healthy food products (Fig. 1b). Participants were recruited by a market research company. The inclusion criteria were being 18 years of age or older and using a computer with internet access to complete the questionnaire. The exclusion criteria included the use of tablets or smartphones, since the size of the products needed to mimic real dimensions, and employment in the food or nutrition field.

The study included representatives of the Brazilian population in terms of sex, geographic region, education and income, who were randomized into four experimental groups: control (without FOPNL), black octagon, black triangle and black magnifying glass. All participants viewed 12 front panels of food labels and answered questions assessing their understanding of nutritional information, perception of healthfulness and purchase intention for the evaluated products, based on a factorial design (3 × 2 × 2): 1) food category (whole-grain cookies, cereal bars and snacks); 2) excess amount of nutrients (one or two nutrients); and 3) nutrition claims (absent or present). The order of presentation followed a Latin square design (Fig. 1b). The combination of factors, including food category, excess nutrient content (added sugars, saturated fat, and sodium), and nutrition claims, as well as their classifications, was developed by the *International Network for Food and Obesity/Non-communicable Diseases (NCDs) Research, Monitoring and Action Support* (INFORMAS).

*Fig. 1.* Experimental design of the study. (a) Allocation of participants into four experimental groups: control (without FOPNL), magnifying glass, octagon, and triangle; (b) stimuli presented: front panels presented in a Latin square design, following the factorial design (3 × 2 × 2): 1) food category (whole-grain cookies, cereal bars, and snacks); 2) excess amount of nutrients (one or two nutrients); and 3) nutritional claims (absent or present); (c) sociodemographic variables; and (d) outcomes evaluated.

A total of 48 front panels of food labels were developed, with 12 for each experimental condition. In addition, all participants provided informed consent to participate in the research. The study was approved by the Research Ethics Committee of the Universidade Federal de Minas Gerais (Plataforma Brasil – CAAE2395020.1.0000.5149) and was conducted between December 2021 and February 2022. Details of the experimental design and procedures have been previously published (14).

The survey questionnaire was divided into four stages. Initially, participants provided personal information, such as responsibility for food purchases, frequency of consumption of the evaluated food products, weight (kg), and height (cm). The sociodemographic variables analyzed were defined based on variables used by the Brazilian Institute of Geography and Statistics (IBGE) (26), including region of residence (Central-West, North, Northeast, South, and Southeast), sex (female and male), age (18–24, 25–34, 35–44, 45–54, and 55 years or older), education (Incomplete Elementary Education, Complete Elementary Education or Incomplete High School, Complete High School or Incomplete Higher Education, Complete Higher Education, and Postgraduate Studies), and income categories, defined as < 2 minimum wages, 2–< 4 minimum wages, 4–< 10 minimum wages, 10–< 20 minimum wages, and ≥ 20 minimum wages, considering the minimum wage for 2021 (US$200). Recruitment quotas were based on the most recent census data from IBGE to obtain a sample representative of the Brazilian population in terms of sex, age, education level, and income (26).

In the second stage, 12 food labels were presented. To assess understanding of the nutritional information, participants were asked the following question regarding product composition: ‘Does this product contain any nutrients in quantities greater than those recommended for a healthy diet?’. Perception of healthfulness was assessed with the question ‘Show how healthy you consider this product to be’, and purchase intention was assessed with the question ‘Show your intention to buy this product’. Participants answered both questions using a seven-point scale. In the fourth stage, participants completed a questionnaire assessing their interest in healthy eating using the *General Health Interest subscale of the Health and Taste Attitude Scales* (HTAS) (27), which was translated into Portuguese (28) and validated.

The results of the third and fourth stages are described in the previously published study (14).

Statistical analyses were performed using the *Statistical Package for the Social Sciences* (SPSS) software, version 20.0, with a significance level of 5%. To assess the probability of correctly understanding nutritional information, a generalized linear mixed model (GLMM) with binary logistic regression was fitted, including sociodemographic variables (region, sex, age, education, and income) as fixed effects and participants as a random effect. The model was adjusted for experimental group (control, octagon, triangle, and magnifying glass), and interest in health, which was assessed in the primary study (14). To assess the mean scores of perception of healthfulness and purchase intention, mixed models of analysis of variance (ANOVA) were performed. Fisher’s post hoc test was used to identify the source of significance. For these models, the same fixed effects, random effect, and adjustment variables as in the previous model were considered. Sample size power for each sociodemographic test was calculated using the pwr.anova.test function from the PWR package in R, with support from the PSSHealth project. Graphs were prepared by the authors using GraphPad Prism software version 8.0.

## Results

The distribution of categories for each sociodemographic variable was consistent with that of the entire Brazilian population, as previously reported in the original study (14). The largest groups within each category were females (51.7%), individuals aged 18–24 years (33.3%), residents of the Southeast region of Brazil (42.2%), individuals with incomplete high school education (38.9%), and those who reported an income of 4–10 minimum wages (55.6%).

**Fig. 2:**
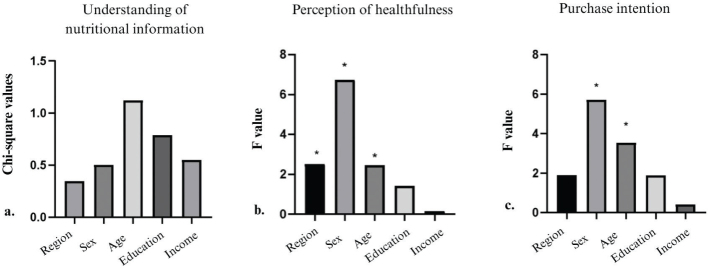
Chi-square and *F*-values and statistical significance of the fixed effects of sociodemographic variables on the three outcomes evaluated. Asterisks denote statistically significant effects (*P* < 0.05). Panel (a) presents the Chi-square statistics estimated from the Generalized Linear Mixed Model for the outcome understanding of nutritional information, while panels (b) and (c) show the test statistics from the ANOVA models for perception of healthfulness and purchase intention, respectively. Higher Chi-square or *F*-values indicate a stronger influence of the sociodemographic variable on the corresponding outcome.

No significant effect of the sociodemographic variables analyzed was observed on the understanding of nutritional information (Fig. 2a). The perception of healthfulness differed according to region, sex, and age (Fig. 2b). Regarding purchase intention, sex and age showed significant effects (Fig. 2c).

**Fig. 3:**
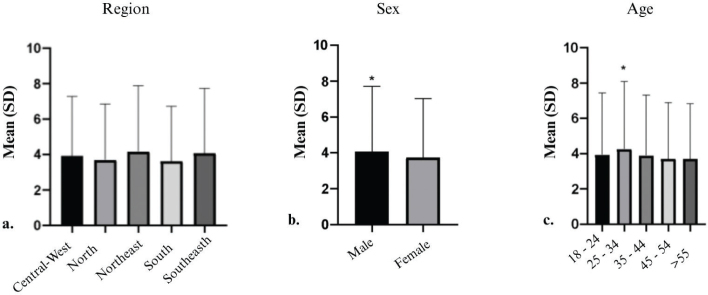
Average scores of healthfulness perception by region (a), sex (b), and age group (c). Note: Only the age group 25–34 years and males differed significantly from the other groups. *P < 0.05 between categories of sociodemographic variables according to the outcome evaluated.

**Fig. 4:**
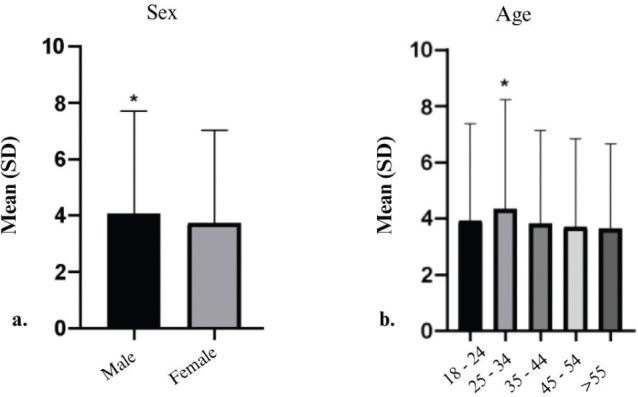
Average scores of purchase intention by sex (a) and age group (b). Note: Only the age group 25–34 years and the male sex differed significantly from the other groups. *P < 0.05 between the ranges of sociodemographic variables according to the outcome evaluated.

Perception of healthfulness was higher among males (Fig. 3b) and among individuals aged 25–34 years (Fig. 3c). Although region showed a significant effect on perception of healthfulness (Fig. 2b), this effect was not confirmed by Fisher’s post hoc test (Fig. 3a). Purchase intention was also higher among males and among individuals aged 25–34 years (Fig. 4).

## Discussion

This study evaluated the effect of sociodemographic variables of Brazilian consumers on the understanding of nutritional information, perception of healthfulness, and intention to purchase food products with FOPNL and nutrition claims. The results showed that the perception of healthfulness and intention to purchase were higher among males and among individuals aged 25–34 years, whereas understanding of nutritional information was not influenced by any of the variables analyzed.

The perception of healthfulness and food choices at the time of purchase can be influenced by several factors. For example, region, income, education, age, and sex may affect the frequency of obtaining and consuming healthy or unhealthy foods (29, 30). In the study by Niven et al. (31), the perception of healthfulness of different foods among the Australian population was analyzed, considering variables such as socioeconomic area, sex, age, and geographic region. The sample included 1,097 adults aged 18–64 years and also included students and experts in the field of nutrition. The results indicated that men and individuals living in areas with lower socioeconomic status had higher perception of healthfulness regarding products that appeared healthier but had unfavorable nutritional composition. Women, students, and participants who were experts in nutrition demonstrated lower perception of healthfulness for unhealthy products, consistent with the findings related to sex in this study.

One possible explanation for the higher levels of perception of healthfulness and purchase intention observed among men is their lower level of health knowledge (32), more limited understanding of labeling, and lower likelihood of being responsible for grocery shopping compared to women (21, 33–35). This may occur because most food-related tasks are typically assigned to women, who also tend to express greater concern about health (31, 36). However, it is important to note that households consisting of single men were not specifically evaluated.

Acton et al. (7) investigated the perception of healthfulness of commercially available beverages among Canadian consumers in relation to different FOPNL models and found no significant effect of sociodemographic variables on the outcomes analyzed. Nevertheless, these results differ from those of this study, as the food products evaluated were not considered supposedly healthy and did not include nutrition claims. Machín et al. (37) reported higher perceptions of healthfulness of ultra-processed foods among the Uruguayan population with lower socioeconomic status. However, the products evaluated did not use the same FOPNL model as in this study, which may have influenced the findings.

Regarding age, participants aged 25–34 years showed higher perception of healthfulness and purchase intention of the evaluated products. A study by Silva et al. (7) demonstrated that Brazilian adults aged 20–29 and 30–39 years had higher consumption of ultra-processed foods. Similar findings were reported by Marchese et al. (38) in a study evaluating young Australians aged 19–30 years. Higher consumption of ultra-processed foods may be related to entry into the job market among this age group and a decline in cooking habits among more recent generations (21), which may be attributed to the greater availability, convenience, palatability, and ease of use of these products (38, 39). Louzada et al. (40) observed that, in Brazil, consumption of ultra-processed foods is inversely proportional to age; that is, as age increases, the presence of these products in the diet tends to decrease. It is possible that the higher consumption of these unhealthy foods among younger individuals, in contrast to older adults, is related to the higher perception of healthfulness and purchase intention observed in this study among individuals aged 25–34 years. In contrast, one possible explanation for differences in perceptions of healthfulness is that older individuals may rely more on accumulated nutrition knowledge, personal health concerns, and established dietary habits when evaluating food products.

In addition, the higher perception observed among younger individuals may be related to the use of heuristics, that is, simple rules used to judge whether a food is healthy. This perception may have occurred since the foods presented in this study appeared to be healthy, mainly due to the images displayed on the labels and the presence of nutrition claims. Both factors may have triggered a heuristic that favors positive information on the label, leading to information bias and reduced attention to less desirable product characteristics (41, 42).

Although understanding of nutritional information did not significantly vary across sociodemographic variables, differences in other outcomes suggest potential inequalities in how labeling influences consumer responses. These findings highlight the importance of ensuring that front-of-pack labeling systems are equally effective across population subgroups.

To the best of our knowledge, this study is the first to evaluate the effect of sociodemographic variables among Brazilian consumers on the understanding and use of different FOPNL systems, including the magnifying glass model recently adopted in Brazil. We also assessed the effect of sociodemographic variables under conditions in which nutrition claims and FOPNL are presented jointly, a practice permitted by Brazilian legislation (43).

Some limitations should be noted. This study includes the online experimental environment, the positioning of nutrition claims on the label mockups, potential response bias resulting from the order of questions in the questionnaire, and the limited number of food categories evaluated. Moreover, the use of an online survey required participants to have access to a computer and adequate reading ability. In addition, given the significant increase in online food purchasing, it is important to understand how consumers interpret nutrition labeling and make food choices in digital shopping environments (44). Since the study required participants to use a computer to ensure proper visualization of the labels, individuals without computer access may have been excluded, potentially limiting the representativeness of the sample. Also, potential effects of sociodemographic factors on the studied outcomes may not have been detected due to insufficient statistical power in the dataset. Assuming a minimum statistical power of 80% and a significance level of 5%, the minimum standardized effect sizes detectable were: 0.10 for sex and 0.225 for age and region, and 0.275 for education level, all of which are considered small effect sizes. However, for income, the minimum detectable effect size was 0.55, corresponding to a large effect size.

## Conclusion

This study evaluated how sociodemographic characteristics influence the understanding of nutritional information, perception of healthfulness, and purchase intention within the specific context of the products, FOPNL models, and nutrition claims tested. Under these conditions, sociodemographic variables did not significantly affect the understanding of nutritional information.

Nonetheless, for the products and labeling formats examined, men and adults aged 25–34 years showed slightly higher perceptions of healthfulness and purchase intention. Although these findings contribute to the evidence base supporting the implementation of the magnifying glass warning symbol in Brazil, they cannot be generalized beyond the conditions tested. Further research is needed to examine these results in real-world shopping environments, with a wider variety of products, label designs, and population groups, including individuals with less digital access.
